# Closing the gap in kidney disease: validating the reporting of Aboriginal and/or Torres Strait Islander identification in a clinical quality registry using linked data

**DOI:** 10.5694/mja2.52613

**Published:** 2025-03-16

**Authors:** Heather J Baldwin, Nicole De La Mata, Grant Sara, Faye McMillan, Brett Biles, Jianyun Wu, Paul Lawton, Stephen McDonald, Angela C Webster

**Affiliations:** ^1^ University of Sydney Sydney NSW; ^2^ Children's Hospital at Westmead Sydney NSW; ^3^ Ministry of Health NSW Government Sydney NSW; ^4^ University of Technology Sydney Sydney NSW; ^5^ Charles Sturt University Albury NSW; ^6^ UNSW Sydney Sydney NSW; ^7^ Charles Darwin University Darwin NT; ^8^ Monash University Melbourne VIC; ^9^ Australia and New Zealand Dialysis and Transplant Registry Adelaide SA; ^10^ Royal Adelaide Hospital Adelaide SA; ^11^ Centre for Renal and Transplant Research Westmead Hospital Sydney NSW

**Keywords:** Kidney transplantation, Renal dialysis, Statistics, Data collection, Social determinants of health, Datasets as topic, Public health, Health services research, Culture

## Abstract

**Objective:**

To examine the accuracy of the Australia and New Zealand Dialysis and Transplant Registry (ANZDATA), the population‐based clinical quality registry for people with kidney failure, in identifying Aboriginal and/or Torres Strait Islander people.

**Design:**

Population‐based cohort study of reporting accuracy.

**Setting:**

New South Wales, 2006–2020.

**Participants:**

Incident kidney failure patients.

**Main outcome measures:**

Sensitivity and specificity of identification of Aboriginal and/or Torres Strait Islander people in ANZDATA compared with identification with Enhanced Reporting of Aboriginality (ERA) methods using linked health datasets.

**Results:**

Of 11 708 patients, 693 (5.9%) were identified as Aboriginal and/or Torres Strait Islander people using ERA methods, with 484 recognised in ANZDATA. Overall ANZDATA sensitivity was 67.0% (95% CI, 63.3–70.5%), with high specificity (99.8%; 95% CI, 99.7–99.9%). Sensitivity was lowest for males (63.8%; 95% CI, 58.7–68.6), people aged under 18 years (45.0%; 95% CI, 23.1–68.5%) or over 65 years (61.7%; 95% CI, 53.8–69.2%), and those with greater socio‐economic advantage (56.6%; 95% CI, 46.6–66.2%), living in major cities (53.8%; 95% CI, 48.0–59.5%) and with no comorbidities (47.7%; 95% CI, 37.0–58.6%). Aboriginal and/or Torres Strait Islander people identified in ANZDATA had lower rates of waitlisting for kidney transplantation (17.8% *v* 25.3%; *P* = 0.016) and receiving a kidney transplant (12.2% *v* 23.1%; *P* < 0.001) and a higher rate of death (56.0% *v* 44.5%; *P* = 0.004) compared with those not recognised in ANZDATA.

**Conclusion:**

Aboriginal and/or Torres Strait Islander people were under‐reported in ANZDATA. There were multiple biases in characteristics and outcomes for people identified in ANZDATA compared with those identified by ERA using linked data. This highlights the importance of data integration as a quality improvement mechanism and identifying barriers to disclosure.

**The known:** Reporting of Aboriginal and/or Torres Strait Islander identity is essential for identifying and acting on health inequity, but reporting may not be accurate.

**The new:** One‐third of Aboriginal and/or Torres Strait Islander people with kidney failure were not identified in the national kidney failure registry. There was systematic bias in characteristics and outcomes for Aboriginal and/or Torres Strait Islander patients with kidney failure who were identified in the Australia and New Zealand Dialysis and Transplant Registry.

**The implications:** Strategies to improve data collection and address barriers may improve accuracy. Routine data linkage could reduce bias in reporting on kidney disease for Aboriginal and/or Torres Strait Islander people.

Accurate identification of Aboriginal and/or Torres Strait Islander people in health data is crucial for measuring service delivery and outcomes, and evaluating policies aimed at closing the gap. However, Aboriginal and/or Torres Strait Islander people are known to be under‐identified in administrative health datasets.^1^, ^2^, ^3^, ^4^, ^5^, ^6^ Validation studies have estimated correct identification of Aboriginal and/or Torres Strait Islander identity in the NSW Admitted Patient Data Collection (APDC), NSW Emergency Department Data Collection (EDDC) and NSW Perinatal Data Collection at between 68% and 84% of records.^5^, ^7^, ^8^

Barriers to recording Aboriginal and/or Torres Strait Islander identity in health data include reluctance of staff to ask the national standard question that has been recommended in guidelines since 2010 — “Are you of Aboriginal or Torres Strait Islander origin?“^4^ — and patient choice not to self‐identify. Under‐identification may result in differences in characteristics or health outcomes for Aboriginal and/or Torres Strait Islander people who are identified compared with those who are not.^5^, ^8^, ^9^, ^10^ Failure to recognise Aboriginal and/or Torres Strait Islander identity may also have direct impact through lack of awareness of a person's cultural identification and needs.

Kidney failure underlies or is associated with 16% of deaths among Aboriginal and/or Torres Strait Islander people.^11^ Kidney transplantation increases quality and length of life compared with dialysis. The Australia and New Zealand Dialysis and Transplant Registry (ANZDATA) is the national clinical quality registry collating information on people receiving dialysis or a kidney transplant from 1964. Aboriginal and/or Torres Strait Islander people are less likely to receive a kidney transplant and are at increased risk of poor post‐transplant outcomes compared with non‐Indigenous people.^12^, ^13^ The National Indigenous Kidney Transplantation Taskforce aims to address evidence and service delivery gaps, facilitate improved access to kidney waitlisting and improve post‐transplantation outcomes.^13^

To address under‐reporting of Aboriginal and Torres Strait Islander identity, the New South Wales Ministry of Health routinely uses the Enhanced Reporting of Aboriginality (ERA) method.^5^ ERA pools information about a person's Aboriginal and/or Torres Strait Islander status from multiple data collections,^5^ and has been demonstrated to substantially correct under‐ascertainment.^2^, ^3^, ^5^, ^14^ Reporting of Aboriginal and Torres Strait Islander identification in ANZDATA has not previously been validated. We linked NSW health data collections to ANZDATA and aimed to use ERA to measure accuracy of reporting of Aboriginal and Torres Strait Islander identity in ANZDATA, and compare characteristics and outcomes for Aboriginal and/or Torres Strait Islander people whose cultural identity was recognised in ANZDATA with characteristics and outcomes for those not recognised.

## Methods

We performed a population‐based retrospective cohort study of people in NSW who received kidney failure treatment (dialysis or transplantation) between 1 July 2006 and 31 December 2020. We linked data collected by ANZDATA to NSW population health data captured in the Mental Health Living Longer (MHLL) program,^15^ and compared recording of Aboriginal and Torres Strait Islander status. We report our study following the Standards for Reporting Diagnostic Accuracy Studies (STARD) guidelines (Supporting Information, STARD 2015 checklist).^16^

### Terminology

We followed the Public Health Association of Australia's *Aboriginal and Torres Strait Islander guide to terminology*.^17^, ^18^ The terms “Indigeneity” and “Aboriginality” have been used only in the context of the national standard question regarding Indigeneity and ERA, which are established, specific methods. We recognise that these terms are not preferred by Aboriginal and/or Torres Strait Islander people and refer readers to the aforementioned guide for terminology guidance.^17^

Information about sex and gender was obtained from ANZDATA, which is collected under the item “gender” (formerly “sex”) and recorded in binary terms as “male” or “female”. We recognise that “sex” usually refers to reporting biological factors, whereas “gender” is usually used to refer to identity and psychosocial or cultural factors. Within the data source, “male” or “female” may relate to sex or gender. Noting this, we use the term “sex” hereafter.

### Data sources

ANZDATA collects information on all patients receiving treatment for kidney failure in Australia and New Zealand. Data are collected in real time and annually on all incident patients, change of treatment modality or location, transplantation, and death (Box 1).^19^ Collected data include demographics, comorbidities, and clinical information relating to kidney disease.

Box 1 Sources of information about Aboriginal and/or Torres Strait Islander identity for ANZDATA and the MHLL cohort*

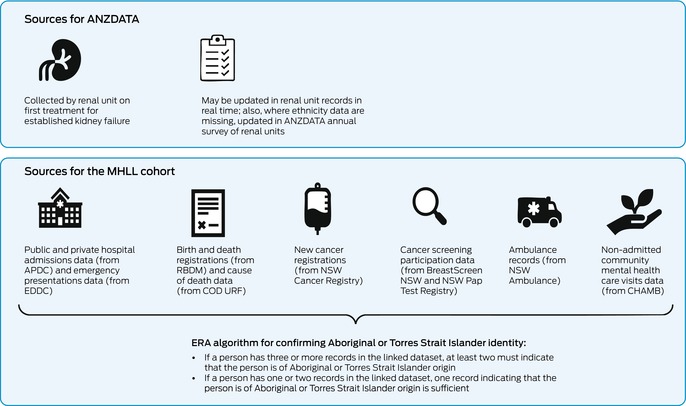

ANZDATA = Australia and New Zealand Dialysis and Transplant Registry; APDC = NSW Admitted Patient Data Collection; CHAMB = Community Mental Health Ambulatory Data Collection; COD URF = Cause of Death Unit Record File; EDDC = NSW Emergency Department Data Collection; ERA = Enhanced Reporting of Aboriginality; MHLL = Mental Health Living Longer; RBDM = NSW Registry of Births, Deaths and Marriages. * The national standard question, used for all sources, is “Are you of Aboriginal or Torres Strait Islander origin?” All people receiving treatment for kidney failure in New South Wales are represented in the MHLL cohort.

Data on ethnicity, and whether patients are of Aboriginal and/or Torres Strait Islander origin, are provided by local health teams and collated by ANZDATA at the time of registration for new patients, or via the annual survey if omitted.^20^ The ANZDATA data item “ethnicity” uses the Australian Bureau of Statistics standard ethnicity classifications.^21^ Aboriginal and/or Torres Strait Islander identity is recorded as 1102 for “Oceanian, Australian Peoples, Australian Aboriginal” and 1104 for “Oceanian, Australian Peoples, Torres Strait Islander”. Before 2014, ethnicity information was recorded as “racial origin” with its own coding system, with options including 2 for “Australian Aboriginal” and 65 for “Torres Strait Islander”. Since 2019, patients have been able to identify a primary and secondary ethnicity. The method for ascertaining ethnicity and the manner in which the standard question on Indigeneity is asked is determined by the renal unit submitting the data.^22^

The linked NSW data assets used for ERA were the APDC, the EDDC, NSW Ambulance data, the Community Mental Health Ambulatory Data Collection, birth and death records from the NSW Registry of Births, Deaths and Marriages, the Australian Bureau of Statistics Cause of Death Unit Record File, the NSW Cancer Registry, BreastScreen NSW and the NSW Pap Test Register (Box 1). A detailed description is available elsewhere.^15^ The cohort includes all NSW residents with a record in at least one of these collections, and is not limited to people who have had contact with mental health services. All people receiving treatment for kidney failure are represented in the APDC.

Since 2012, the national standard question regarding Indigeneity has been used to collect information on Aboriginal and Torres Strait Islander identity in all NSW Health services.^4^, ^22^, ^23^, ^24^ Reporting of self‐identification is enhanced using the standard NSW Health ERA algorithm.^14^ According to the ERA algorithm, Aboriginal and/or Torres Strait Islander identity can be confirmed in two ways: a person who has three or more independent records within the linked datasets must have at least two that indicate that the person is of Aboriginal and/or Torres Strait Islander origin, while a person who has records in one or two datasets must have at least one indicating that the person is of Aboriginal and/or Torres Strait Islander origin (Box 1).^14^

Data from ANZDATA and the MHLL program were probabilistically linked by the Centre for Health Record Linkage based on personal identifiers.^25^ Rates of false positive and missed links are each estimated to be about 5 per 1000.^25^

### Study population

We included all incident patients who received treatment for kidney failure in NSW between 1 July 2006 and 31 December 2020, based on their inclusion in ANZDATA.

### Patient characteristics and outcomes

Information on patient demographics, clinical characteristics and kidney disease outcomes, and physical comorbidities were obtained from ANZDATA, while data on mental health care were obtained from MHLL datasets. Both sources were used to ascertain patient death. Age, comorbidities, remoteness and socio‐economic status were defined at the start of kidney replacement therapy (KRT). Severe and persistent mental illness was defined as pre‐existing if an inpatient or mental health community care episode was recorded within one year before or after starting KRT. Socio‐economic status was calculated using the Socio‐Economic Indexes for Areas Index of Relative Socio‐economic Disadvantage.^26^ Remoteness was calculated using the Australian Statistical Geography Standard Remoteness Structure.^27^ Late referral was defined as the patient having their first assessment by the renal unit less than three months before they first received KRT. Patients with no reported ethnicity were counted as non‐Indigenous.

### Statistical analysis

Reporting of Aboriginal and/or Torres Strait Islander self‐identification in ANZDATA was compared with ERA status from the MHLL linkage program as the reference standard. Sensitivity, specificity, positive predictive values (PPVs) and negative predictive values (NPVs) were reported with exact confidence intervals for the entire study cohort and by patient characteristics. Differences in sensitivity by source were statistically tested using McNemar's test. Differences in patient characteristics, outcomes by source of reporting, and sensitivity between patient groups were statistically tested using χ2 tests. Sensitivity, specificity, PPVs and NPVs were also calculated for identification in ANZDATA augmented with ERA from the APDC, compared with ERA from all MHLL datasets as the reference standard, to assess whether linkage to a single dataset would improve ascertainment. Trends in sensitivity were examined over time using linear regression and the Cochran–Armitage test for trends. Records with missing data were excluded from analysis of that variable (Supporting Information, Supplementary Table 1). Analysis was performed in SAS 9.3 (SAS Institute).

## Study governance, ethics approval and authorship

Our study was identified as a priority by the MHLL Aboriginal Sovereign Steering Committee. The committee has been involved in the study since its inception and throughout the processes of designing the study, interpreting the results, and drafting the manuscript for this article. Two of us (FM and BB) are members of this committee.

This study received ethics approval from the Aboriginal Health and Medical Research Council of NSW Human Research Ethics Committee (1564/19) and the NSW Population and Health Services Research Ethics Committee (2019/ETH01620).

## Results

Of the 11 772 people who commenced KRT in NSW between 2006 and 2020, 11 708 (99.5%) had a linked record in at least one of the nine linked health data collections and were included (Box 2). A total of 693 people were identified as Aboriginal and/or Torres Strait Islander using ERA (5.9%). ANZDATA recognised 484 (4.1% of 11 708), including 20 people not recognised using ERA (Supporting Information, Supplementary Table 2).

Box 2 **Flow diagram of participants included in the study, identified from ANZDATA and linked to nine NSW administrative population health datasets from the MHLL linkage program**

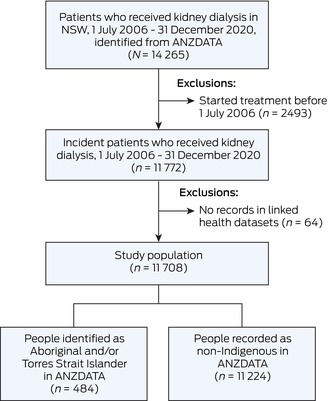

ANZDATA = Australia and New Zealand Dialysis and Transplant Registry; MHLL = Mental Health Living Longer; NSW = New South Wales.

Characteristics of people recognised as Aboriginal and/or Torres Strait Islander are shown by source of reporting in Box 3. A higher proportion of females were identified in ANZDATA compared with those in ERA alone, although the difference was not statistically significant (47.5% *v* 40.2%; *P* = 0.07). There was a significantly lower proportion of people aged under 18 years and over 65 years captured in ANZDATA compared with ERA alone, as well as people with less socio‐economic disadvantage and people living in major cities. There were lower rates of physical comorbidities in ANZDATA (eg, 9.1% with no comorbidities in ANZDATA compared with 20.1% for ERA only; *P* < 0.001) and marginally lower rates of overweight or obesity in the ERA only group (68.1% *v* 74.6%; *P* = 0.049). For cause of kidney failure, there were significantly higher rates of glomerulonephritis or immunoglobulin A nephropathy and polycystic kidney and lower rates of diabetes for the ERA only group. There were no significant differences in rates of home dialysis, late referral, smoking or mental illness for the group captured in ANZDATA compared with those in the ERA only group.

Box 3 **Patient and clinical characteristics of NSW Aboriginal and/or Torres Strait Islander patients with kidney failure, stratified by recognition in ANZDATA compared with ERA using linked NSW administrative data**

**Table:** 

	Number (%) of patients*			
Characteristic	ANZDATA (*n* = 484)	ERA only (*n* = 229)	Total (*n* = 713)	*P* ^†^
Sex				0.07
Female	230 (47.5%)	92 (40.2%)	322 (45.2%)	
Male	254 (52.5%)	137 (59.8%)	391 (54.8%)	
Age (years)				0.028
< 18	9 (1.9%)	11 (4.8%)	20 (2.8%)	
18–44	89 (18.4%)	44 (19.2%)	133 (18.7%)	
45–64	281 (58.1%)	112 (48.9%)	393 (55.1%)	
≥ 65	105 (21.7%)	62 (27.1%)	167 (23.4%)	
Socio‐economic status^26^				0.009
Q1 — most disadvantaged	182 (37.6%)	58 (25.3%)	240 (33.7%)	
Q2	153 (31.6%)	90 (39.3%)	243 (34.1%)	
Q3	79 (16.3%)	33 (14.4%)	112 (15.7%)	
Q4	47 (9.7%)	31 (13.5%)	78 (10.9%)	
Q5 — least disadvantaged	21 (4.3%)	15 (6.6%)	36 (5.0%)	
Remoteness^27^				
Major city	179 (37.0%)	140 (61.1%)	319 (44.7%)	< 0.001
Outside city	304 (62.8%)	88 (38.4%)	392 (55.0%)	
Body mass index (kg/m^2^)				0.049
Underweight or normal (< 25.0)	112 (23.1%)	69 (30.1%)	181 (25.4%)	
Overweight or obese (≥ 25.0)	361 (74.6%)	156 (68.1%)	517 (72.5%)	
Smoking status				0.18
Current	113 (23.3%)	54 (23.6%)	167 (23.4%)	
Former	224 (46.3%)	91 (39.7%)	315 (44.2%)	
Never	147 (30.4%)	84 (36.7%)	231 (32.4%)	
Mental illness^‡^				0.20
Severe and persistent mental illness	38 (7.9%)	22 (9.6%)	60 (8.4%)	
Moderate mental illness	20 (4.1%)	< 5 (< 2.2%)	24 (3.4%)	
Duration of mental illness				0.71
0–2 years	88 (18.2%)	40 (17.5%)	128 (18.0%)	
> 2 years	51 (10.5%)	26 (11.4%)	77 (10.8%)	
Mental health care				0.034
Any inpatient	66 (13.6%)	21 (9.2%)	87 (12.2%)	
Ambulant only	73 (15.1%)	45 (19.7%)	118 (16.5%)	
Pre‐existing comorbidity^‡^				
Any diabetes	342 (70.7%)	105 (45.9%)	447 (62.7%)	< 0.001
Cardiovascular and cerebrovascular diseases^§^	318 (65.7%)	113 (49.3%)	431 (60.4%)	< 0.001
Another comorbidity^¶^	202 (41.7%)	71 (31.0%)	273 (38.3%)	0.006
Comorbidity count**				< 0.001
0	44 (9.1%)	46 (20.1%)	90 (12.6%)	
1	97 (20.0%)	72 (31.4%)	169 (23.7%)	
2	159 (32.9%)	62 (27.1%)	221 (31.0%)	
≥ 3	184 (38.0%)	49 (21.4%)	233 (32.7%)	
Cause of kidney disease				< 0.001
Diabetes	294 (60.7%)	84 (36.7%)	378 (53.0%)	
Hypertension or renal artery disease	63 (13.0%)	22 (9.6%)	85 (11.9%)	
GN or IgA nephropathy	65 (13.4%)	48 (21.0%)	113 (15.8%)	
Polycystic kidney	9 (1.9%)	16 (7.0%)	25 (3.5%)	
Other^††^	53 (11.0%)	59 (25.8%)	112 (15.7%)	
Initial KRT type				0.11
Haemodialysis	358 (74.0%)	171 (74.7%)	529 (74.2%)	
Peritoneal dialysis	126 (26.0%)	56 (24.5%)	182 (25.5%)	
Transplant	0	< 5 (< 2.2%)	< 5 (< 0.7%)	
Place of dialysis				0.71
In centre	356 (73.6%)	170 (74.2%)	526 (73.8%)	
At home	128 (26.4%)	57 (24.9%)	185 (25.9%)	
Late referral^‡‡^	111 (22.9%)	44 (19.2%)	155 (21.7%)	0.25

ANZDATA = Australia and New Zealand Dialysis and Transplant Registry; ERA = Enhanced Reporting of Aboriginality; GN = glomerulonephritis; IgA = immunoglobulin A; KRT = kidney replacement therapy; NSW = New South Wales; Q = quintile. * Counts of less than 5 are not reported in accordance with privacy restrictions. † χ2 test. ‡ Pre‐existing at the start of KRT. § Coronary artery disease, cerebrovascular disease, peripheral vascular disease. ¶ Chronic lung disease, history of cancer, hepatitis C. ** Any diabetes, coronary artery disease, cerebrovascular disease, peripheral vascular disease, chronic lung disease, history of cancer and/or hepatitis C, pre‐existing at the start of KRT. †† Other causes of kidney disease included: drug and heavy metal toxicity (*n* = 16, 14.3% of 112); congenital abnormalities of the kidney and urinary tract (*n* = 27, 24.1% of 112); obstructions (*n* = 6, 5.4% of 112); cancer (*n* = 4, 3.6% of 112), inherited metabolic disorders of the kidney (*n* = 3, 2.7% of 112); uncertain diagnosis (*n* = 38, 33.9% of 112); and other non‐specified condition (*n* = 18, 16.1% of 112). ‡‡ Less than three months before commencing KRT. Note: due to missing data, not all values add to column totals.

Overall ANZDATA sensitivity was 67.0% (95% CI, 63.3–70.5%; *P* < 0.001). In terms of sex and age, the lowest sensitivity was among people aged under 18 years (45.0%; 95% CI, 23.1–68.5%) and highest sensitivity was among female patients (70.8%; 95% CI, 65.4–75.8%) (Box 4). Higher sensitivity was associated with lower socio‐economic status, living outside of a major city, inpatient mental health care, and multimorbidity. Specificity was high across all groups, at 99.8% (95% CI, 99.7–99.9%) overall. Similarly, PPVs were high, at 95.9% (95% CI, 93.7–97.5%) overall; PPV was lowest among the least socio‐economically disadvantaged (88.2%; 95% CI, 78.1–94.8%) and 100% among people aged under 18 years (95% CI, 66.4–100%), those with moderate mental illness (95% CI, 83.2–100%), and those accessing inpatient mental health care (95% CI, 94.6–100%). NPV was lowest among people aged under 18 years (93.3%; 95% CI, 88.2–96.6%) and highest among people with moderate mental illness (99.3%; 95% CI, 98.3– 99.8%). There was no significant change in sensitivity over time (linear regression: β = 0.21, standard error = 0.51, *P* = 0.694; Cochran‐Armitage: *P* = 0.537 [Box 5]). When self‐identification data from ANZDATA was augmented with ERA from the APDC and compared with ERA from the MHLL linkage program, overall sensitivity increased to 94.8% (95% CI, 92.9–96.3% [Supporting Information, Supplementary Table 3]).

Box 4 **Accuracy of reporting of Aboriginal and/or Torres Strait Islander identification in ANZDATA compared with ERA using linked NSW administrative data as the reference standard**

**Table:** 

Characteristic	Number of patients identified in ANZDATA	Number of patients identified using ERA	Sensitivity (95% CI)	Specificity (95% CI)	Positive predictive value (95% CI)	Negative predictive value (95% CI)
Overall	484	693	67.0% (63.3–70.5%)	99.8% (99.7– 99.9%)	95.9% (93.7– 97.5%)	98.0% (97.7– 98.2%)
Sex						
Female	230	315	70.8% (65.4–75.8%)	99.8% (99.6–99.9%)	97.0% (93.8–98.8%)	97.7% (97.2–98.2%)
Male	254	378	63.8% (58.7–68.6%)	99.8% (99.7–99.9%)	94.9% (91.4–97.2%)	98.1% (97.8–98.4%)
Age (years)						
< 18	9	20	45.0% (23.1–68.5%)	100.0% (97.6–100.0%)	100.0% (66.4–100.0%)	93.3% (88.2–96.6%)
18–44	89	129	65.9% (57.0–74.0%)	99.7% (99.3–99.9%)	95.5% (88.9–98.8%)	96.9% (95.8–97.7%)
45–64	281	382	70.7% (65.8–75.2%)	99.7% (99.5–99.9%)	96.1% (93.1–98.0%)	97.2% (96.6–97.7%)
≥ 65	105	162	61.7% (53.8–69.2%)	99.9% (99.8–100.0%)	95.2% (89.2–98.4%)	98.9% (98.6–99.2%)
Socio‐economic status^26^ *						
Most disadvantaged (Q1 and Q2)	335	473	68.7% (64.3–72.9%)	99.8% (99.6–99.9%)	97.0% (94.6–98.6%)	97.2% (96.7–97.6%)
Average (Q3)	79	110	70.0% (60.5–78.4%)	99.9% (99.6–100.0%)	97.5% (91.2–99.7%)	98.2% (97.5–98.7%)
Least disadvantaged (Q4 and Q5)	68	106	56.6% (46.6–66.2%)	99.8% (99.6–99.9%)	88.2% (78.1–94.8%)	98.9% (98.5–99.2%)
Remoteness^27^						
Major city	179	303	53.8% (48.0–59.5%)	99.8% (99.7–99.9%)	91.1% (85.9–94.8%)	98.4% (98.1–98.7%)
Outside major city	304	388	77.3% (72.8–81.4%)	99.8% (99.5–100.0%)	98.7% (96.7–99.6%)	96.3% (95.4–97.0%)
Mental illness^†^						
Severe and persistent	38	59	62.7% (49.1–75.0%)	99.8% (98.7–100.0%)	97.4% (86.2–99.9%)	94.9% (92.4–96.8%)
Moderate mental illness	20	24	83.3% (62.6–95.3%)	100.0% (99.4–100.0%)	100.0% (83.2–100.0%)	99.3% (98.3–99.8%)
None	426	610	66.7% (62.8–70.5%)	99.8% (99.7–99.9%)	95.5% (93.1–97.3%)	98.0% (97.7–98.3%)
Mental health care accessed						
Any inpatient	66	87	75.9% (65.5–84.4%)	100.0% (99.3–100.0%)	100.0% (94.6–100.0%)	96.4% (94.6–97.8%)
Community only	73	115	60.9% (51.3–69.8%)	99.8% (99.4–100.0%)	95.9% (88.5–99.1%)	96.9% (95.9–97.8%)
Mental illness duration (years)						
0–2 years	88	126	68.3% (59.4–76.3%)	99.9% (99.5–100.0%)	97.7% (92.0–99.7%)	97.3% (96.3–98.1%)
> 2 years	51	76	65.8% (54.0–76.3%)	99.8% (99.0–100.0%)	98.0% (89.6–100.0%)	95.5% (93.4–97.0%)
Pre‐existing comorbidity^†^						
Diabetes	342	434	75.8% (71.5–79.8%)	99.8% (99.6–99.9%)	96.2% (93.6–98.0%)	98.0% (97.6–98.4%)
Cardiovascular and cerebrovascular diseases^‡^	318	419	73.0% (68.5–77.2%)	99.8% (99.6–99.9%)	96.2% (93.5–98.0%)	98.0% (97.6–98.4%)
Other^§^	202	265	73.2% (67.4–78.4%)	99.8% (99.5–99.9%)	96.0% (92.3–98.3%)	98.0% (97.4–98.4%)
Comorbidity count^¶^						
0	44	88	47.7% (37.0–58.6%)	99.9% (99.7–100.0%)	95.5% (84.5–99.4%)	97.9% (97.2–98.5%)
1–2	256	377	64.5% (59.4–69.3%)	99.8% (99.7–99.9%)	94.9% (91.5–97.3%)	98.1% (97.8–98.4%)
≥ 3	184	228	78.5% (72.6–83.7%)	99.7% (99.4–99.9%)	97.3% (93.8–99.1%)	97.4% (96.5–98.0%)

ANZDATA = Australia and New Zealand Dialysis and Transplant Registry; ERA = Enhanced Reporting of Aboriginality; NSW = New South Wales; Q = quintile. * Socio‐economic status quintiles 1 and 2 were grouped together, and quintiles 4 and 5 were grouped together. † Pre‐existing at start of kidney replacement therapy. ‡ Coronary artery disease, cerebrovascular disease, peripheral vascular disease. § Chronic lung disease, history of cancer, hepatitis C. ¶ Any diabetes, coronary artery disease, cerebrovascular disease, peripheral vascular disease, chronic lung disease, history of cancer and/or hepatitis C, pre‐existing at the start of kidney replacement therapy. Note: due to missing data, not all values add to column totals.

Box 5 Sensitivity and specificity of reporting of Aboriginal and/or Torres Strait Islander self‐identification in ANZDATA over time compared with ERA from linked NSW administrative data as the reference standard*

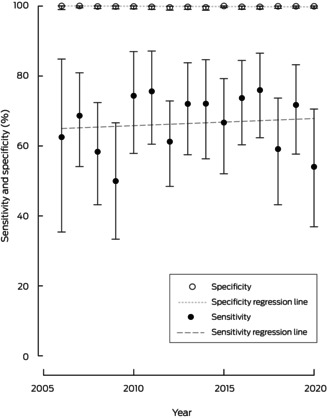

ANZDATA = Australia and New Zealand Dialysis and Transplant Registry; ERA = Enhanced Reporting of Aboriginality; NSW = New South Wales. * Errors bars represent 95% CIs.

In order to examine the robustness of these findings, we evaluated the accuracy of ANZDATA in identifying Aboriginal and/or Torres Strait Islander people compared with the ERA algorithm using APDC alone, and found an overall sensitivity of 70.5% (95% CI, 66.8–74.0%) and PPV of 95.2% (95% CI, 93.0–97.0%), with similar accuracy when analysed by patient and characteristics (Supporting Information, Supplementary Table 4). Further, accuracy of ANZDATA was similar when compared to an algorithm that identified Aboriginal and/or Torres Strait Islander people using only the most recent APDC record for each patient (Supporting Information, Supplementary Table 5).

Outcomes for people who identified as Aboriginal and/or Torres Strait Islander by source of reporting are shown in Box 6. People not recognised by ANZDATA but recognised by ERA alone as Aboriginal and/or Torres Strait Islander had better outcomes. For the ERA only group, there were significantly higher rates of waitlisting (19.7% *v* 11.4% within 2 years; *P* = 0.004), the rate of transplantation within 2 years was more than three times higher (10.9% *v* 3.1%; *P* < 0.001), and the rate of death within 5 years was lower (27.9% *v* 38.0%; *P* = 0.008).

Box 6 **Outcomes for NSW Aboriginal and/or Torres Strait Islander people identified using ANZDATA compared with outcomes for those identified by ERA using linked NSW administrative data as the reference standard**

**Table:** 

	Number (%) of patients			
Outcome	ANZDATA (*n* = 484)	ERA only (*n* = 229)	Total (*n* = 713)	P*
Waitlisted for kidney transplantation^†^				
During study period	86 (17.8%)	58 (25.3%)	144 (20.2%)	0.016
Within 2 years of starting KRT	55 (11.4%)	45 (19.7%)	100 (14.0%)	0.004
Within 5 years of starting KRT	77 (15.9%)	54 (23.6%)	131 (18.4%)	0.017
Received kidney transplant				
During study period	59 (12.2%)	53 (23.1%)	112 (15.7%)	< 0.001
Within 2 years of starting KRT	15 (3.1%)	25 (10.9%)	40 (5.6%)	< 0.001
Within 5 years of starting KRT	39 (8.1%)	40 (17.5%)	79 (11.1%)	< 0.001
Died				
During study period	271 (56.0%)	102 (44.5%)	373 (52.3%)	0.004
Within 2 years of starting KRT	78 (16.1%)	25 (10.9%)	103 (14.4%)	0.07
Within 5 years of starting KRT	184 (38.0%)	64 (27.9%)	248 (34.8%)	0.008

ANZDATA = Australia and New Zealand Dialysis and Transplant Registry; ERA = Enhanced Reporting of Aboriginality; KRT = kidney replacement therapy; NSW = New South Wales. * χ2 test. † Refers to first active waitlisting.

## Discussion

In this study, we evaluated reporting of Aboriginal and Torres Strait Islander self‐identification in ANZDATA against a reference standard comprising data from multiple administrative datasets. We found evidence of under‐recognition of Aboriginal and/or Torres Strait Islander people, and systematic bias in that under‐recording. We found that one‐third of Aboriginal and/or Torres Strait Islander people identified using the ERA approach were not recognised in ANZDATA. Aboriginal and/or Torres Strait Islander people not recognised by ANZDATA were more often male, children or older adults, although the difference between males and females was not statistically significant. They were more likely to have higher socio‐economic advantage, dwell in urban areas and be healthier. They also had better outcomes for kidney failure health services than those who were recognised in ANZDATA; they had better access to transplantation, and reduced mortality. Aboriginal and Torres Strait Islander identity was under‐ascertained in ANZDATA with an overall sensitivity of 67.0%, which has not changed over time. Where ANZDATA recognised a person as being Aboriginal and/or Torres Strait Islander, this was accurate, shown by high specificity and PPV (99.8% and 95.9% respectively).

Our findings emphasise a known problem of under‐reporting of Aboriginal and Torres Strait Islander identity when relying on a single administrative data collection.^1^, ^2^, ^3^, ^4^, ^5^, ^6^, ^28^ The rate of ascertainment that we observed in our study is similar to that reported for the NSW Perinatal Data Collection, which has been estimated at between 68% and 71%,^7^, ^8^ and lower than that reported for death registrations in the NSW Registry of Births, Deaths and Marriages at 76%,^3^ the NSW EDDC at 77%^14^ and the NSW APDC at 84–85%.^5^, ^14^ Systematic bias in patient characteristics and outcomes for Aboriginal and/or Torres Strait Islander people identified by ANZDATA compared with those who were not is consistent with findings of previous research, which have demonstrated lower admission and mortality rates for cardiovascular and injury outcomes,^9^ lower rates of diabetes,^8^ and better child development outcomes^10^ for those identified using enhanced methods.

There are several reasons for differences in reporting of Aboriginal and/or Torres Strait Islander self‐identification. Ethnicity and cultural identity information is collected by ANZDATA once, when data are entered into the registry system, and updated using the annual survey only if data are missing. In comparison, ERA uses information sourced from multiple datasets and multiple records within datasets, increasing opportunities for self‐identification.

Barriers to self‐identification are complex and varied and may include racism in medical care and society more generally. Staff attitudes towards collecting Aboriginal and Torres Strait Islander identity information have been shown to affect likelihood of asking, use of the national standard question and accuracy of the data.^4^ Aboriginal and/or Torres Strait Islander people have the right to choose when and to whom they self‐identify, and past experiences including whether care has been culturally safe are important factors in this choice. These factors may be related to the differences in sensitivity by patient characteristics that we observed in our study, as they may affect self‐identification of patient groups in different ways. Previous studies on accuracy of reporting in unlinked administrative datasets without enhancement have produced similar findings, including lower sensitivity for males,^9^ children^10^ and older adults,^5^, ^8^, ^9^ people who are less socio‐economically disadvantaged,^8^ and people living in more urban settings.^5^, ^8^, ^9^, ^29^ This geographic association suggests that variation in environment and practices among renal units may play a role. Reporting of self‐identification for Aboriginal and/or Torres Strait Islander people has been previously shown to vary between hospitals and local health districts.^5^ Likelihood to be identified in ANZDATA was higher for people with pre‐existing comorbidities, and was highest for people with multimorbidity (three or more comorbidities) and people who received inpatient mental health care. This suggests that more experience with NSW health services may positively affect a person's choice to self‐identify.

Our findings highlight the importance of continued efforts to improve data collection. National guidelines were released in 2010 to improve the collection and recording of Aboriginal and/or Torres Strait Islander status in health datasets,^4^ and the standard question regarding Indigeneity has been mandatory in all NSW health services since 2012.^30^ Despite the first 6 years of data in our study being collected before this change, and contrary to others’ findings that propensity to self‐identify has increased over time,^31^ we found no significant increase in sensitivity over time in ANZDATA. Standardising the way in which the national standard question is asked across renal units, and inclusion of a preamble, may help to improve sensitivity and consistency within the ANZDATA data collection.^4^ Our findings also underscore the importance of education and training for renal unit staff about how to ask the standard question regarding Indigeneity, the reasons for collecting this information, and the importance of asking all patients.^2^ Clinical care in line with recent guidelines for culturally safe kidney care should reduce barriers to disclosure.^32^ Further, routine linkage to external data sources such as those used in our study to enhance reporting would vastly improve ascertainment and reduce bias in research and reporting on kidney health for Aboriginal and/or Torres Strait Islander people. We have shown that sensitivity could be increased to 95% via linkage solely to the NSW APDC.

Strengths of our study include that it was population based, including all people on dialysis in NSW, where one‐third of Australia's Aboriginal population resides,^33^ and the large number of linked datasets and records included in the ERA algorithm.

### Limitations

A limitation of our study is that ERA, while demonstrated to greatly improve accuracy on self‐identification,^2^, ^3^, ^5^, ^8^, ^9^, ^14^ is an imperfect reference standard. There is likely to be some under‐reporting of Aboriginal and Torres Strait Islander identity using ERA, with some people not identified on any of their linked records.^14^ This would result in some over‐estimation of sensitivity in our study. However, the use of nine linked datasets spanning 15 years provided a large number of linked records for maximising the potential of enhancement in our study.

## Conclusion

We found that ANZDATA under‐reported the number of patients who are likely to be of Aboriginal and/or Torres Strait Islander origin, with one‐third of Aboriginal and/or Torres Strait Islander people not recognised as such. We observed systematic bias in demographic and clinical characteristics and poorer outcomes for Aboriginal and Torres Strait Islander patients with kidney failure reported in ANZDATA compared with those not reported. These findings are important and highly relevant to initiatives for improving the health of Aboriginal and/or Torres Strait Islander people. They also support more widespread integration of data to improve equity metrics, including ethnicity and cultural identity in governance of health service design and delivery. Given the disproportionate burden of end‐stage kidney disease in Aboriginal and/or Torres Strait Islander people, such measures are vital for understanding the impact of health programs intended to support wellness and survival, improve outcomes and access to transplantation, and move towards closing the gap in kidney disease.

### Data sharing

The data for this study will not be shared, as we do not have permission from the data custodians or ethics approval to do so. Data may be available upon request from the data custodians.

### Open access

Open access publishing facilitated by The University of Sydney, as part of the Wiley – the University of Sydney agreement via the Council of Australian University Librarians.

### Competing interests

No relevant disclosures.

## Supporting information


Supplementary tables
