# Supplementary material for: Closing the gap in kidney disease: validating the reporting of Aboriginal and/or Torres Strait Islander identification in a clinical quality registry using linked data
Source: Med J Aust. 2025 Mar 16;222(5):240–8. doi: 10.5694/mja2.52613 (PMC11910948; doi:10.5694/mja2.52613)
Supplement: Supplementary file 1 — Supplementary tables [file MJA2-222-240-s001.pdf]

MJA
The Medical Journal of Australia

Supporting Information

Supplementary material

This appendix was part of the submitted manuscript and has been peer reviewed.
It is posted as supplied by the authors.

Appendix to: Baldwin H, De La Mata N, Sara G, et al. Closing the gap in kidney disease: validating reporting of Aboriginal and/or Torres Strait Islander identification in a clinical quality registry using linked data. *Med J Aust* 2025; doi: 10.5694/mja2.52613.

CC BY SA

STARD 2015 checklist

Section & Topic	No	Item	Reported on page #
**TITLE OR ABSTRACT**			
	**1**	Identification as a study of diagnostic accuracy using at least one measure of accuracy (such as sensitivity, specificity, predictive values, or AUC)	2
**ABSTRACT**			
	**2**	Structured summary of study design, methods, results, and conclusions (for specific guidance, see STARD for Abstracts)	2
**INTRODUCTION**			
	**3**	Scientific and clinical background, including the intended use and clinical role of the index test	4
	**4**	Study objectives and hypotheses	4
**METHODS**			
Study design	**5**	Whether data collection was planned before the index test and reference standard were performed (prospective study) or after (retrospective study)	5
Participants	**6**	Eligibility criteria	6
	**7**	On what basis potentially eligible participants were identified (such as symptoms, results from previous tests, inclusion in registry)	6
	**8**	Where and when potentially eligible participants were identified (setting, location and dates)	5,6
	**9**	Whether participants formed a consecutive, random or convenience series	6
Test methods	**10a**	Index test, in sufficient detail to allow replication	5
	**10b**	Reference standard, in sufficient detail to allow replication	5,6
	**11**	Rationale for choosing the reference standard (if alternatives exist)	4
	**12a**	Definition of and rationale for test positivity cut-offs or result categories of the index test, distinguishing pre-specified from exploratory	5
	**12b**	Definition of and rationale for test positivity cut-offs or result categories of the reference standard, distinguishing pre-specified from exploratory	5,6
	**13a**	Whether clinical information and reference standard results were available to the performers/readers of the index test	5
	**13b**	Whether clinical information and index test results were available to the assessors of the reference standard	6
Analysis	**14**	Methods for estimating or comparing measures of diagnostic accuracy	6
	**15**	How indeterminate index test or reference standard results were handled	6
	**16**	How missing data on the index test and reference standard were handled	6
	**17**	Any analyses of variability in diagnostic accuracy, distinguishing pre-specified from exploratory	6
	**18**	Intended sample size and how it was determined	N/A (population based study)
**RESULTS**			
Participants	**19**	Flow of participants, using a diagram	Figure 2
	**20**	Baseline demographic and clinical characteristics of participants	14 (table 1)
	**21a**	Distribution of severity of disease in those with the target condition	N/A
	**21b**	Distribution of alternative diagnoses in those without the target condition	N/A
	**22**	Time interval and any clinical interventions between index test and reference standard	N/A
Test results	**23**	Cross tabulation of the index test results (or their distribution) by the results of the reference standard	Supplementary Table 2
	**24**	Estimates of diagnostic accuracy and their precision (such as 95% confidence intervals)	Table 2
	**25**	Any adverse events from performing the index test or the reference standard	N/A
**DISCUSSION**			
	**26**	Study limitations, including sources of potential bias, statistical uncertainty, and generalisability	9
	**27**	Implications for practice, including the intended use and clinical role of the index test	9,10
**OTHER INFORMATION**			

CC BY SA

	**28**	Registration number and name of registry	N/A
	**29**	Where the full study protocol can be accessed	N/A
	**30**	Sources of funding and other support; role of funders	10

STARD 2015

AIM

STARD stands for “Standards for Reporting Diagnostic accuracy studies”. This list of items was developed to contribute to the completeness and transparency of reporting of diagnostic accuracy studies. Authors can use the list to write informative study reports. Editors and peer-reviewers can use it to evaluate whether the information has been included in manuscripts submitted for publication.

EXPLANATION

A **diagnostic accuracy study** evaluates the ability of one or more medical tests to correctly classify study participants as having a **target condition.** This can be a disease, a disease stage, response or benefit from therapy, or an event or condition in the future. A medical test can be an imaging procedure, a laboratory test, elements from history and physical examination, a combination of these, or any other method for collecting information about the current health status of a patient.

The test whose accuracy is evaluated is called **index test.** A study can evaluate the accuracy of one or more index tests. Evaluating the ability of a medical test to correctly classify patients is typically done by comparing the distribution of the index test results with those of the **reference standard**. The reference standard is the best available method for establishing the presence or absence of the target condition. An accuracy study can rely on one or more reference standards.

If test results are categorized as either positive or negative, the cross tabulation of the index test results against those of the reference standard can be used to estimate the **sensitivity** of the index test (the proportion of participants *with* the target condition who have a positive index test), and its **specificity** (the proportion *without* the target condition who have a negative index test). From this cross tabulation (sometimes referred to as the contingency or “2x2” table), several other accuracy statistics can be estimated, such as the positive and negative **predictive values** of the test. Confidence intervals around estimates of accuracy can then be calculated to quantify the statistical **precision** of the measurements.

If the index test results can take more than two values, categorization of test results as positive or negative requires a **test positivity cut-off**. When multiple such cut-offs can be defined, authors can report a receiver operating characteristic (ROC) curve which graphically represents the combination of sensitivity and specificity for each possible test positivity cut-off. The **area under the ROC curve** informs in a single numerical value about the overall diagnostic accuracy of the index test.

The **intended use** of a medical test can be diagnosis, screening, staging, monitoring, surveillance, prediction or prognosis. The **clinical role** of a test explains its position relative to existing tests in the clinical pathway. A replacement test, for example, replaces an existing test. A triage test is used before an existing test; an add-on test is used after an existing test.

Besides diagnostic accuracy, several other outcomes and statistics may be relevant in the evaluation of medical tests. Medical tests can also be used to classify patients for purposes other than diagnosis, such as staging or prognosis. The STARD list was not explicitly developed for these other outcomes, statistics, and study types, although most STARD items would still apply.

DEVELOPMENT

This STARD list was released in 2015. The 30 items were identified by an international expert group of methodologists, researchers, and editors. The guiding principle in the development of STARD was to select items that, when reported, would help readers to judge the potential for bias in the study, to appraise the applicability of the study findings and the validity of conclusions and recommendations. The list represents an update of the first version, which was published in 2003.

More information can be found on http://www.equator-network.org/reporting-guidelines/stard.

CC BY SA

Supplementary tables

Supplementary Table 1. Distribution of missing data (n = 11,708)

Variable	Number of missing records	%
Ethnicity	193	1.6%
Sex	0	0%
Age	0	0%
Socio-economic status	51	0.4%
Remoteness	51	0.4%
Body mass index	274	2.3%
Smoking	140	1.2%
Chronic lung disease	26	0.2%
Coronary artery disease	24	0.2%
Peripheral vascular disease	23	0.2%
Cerebrovascular disease	23	0.2%
Diabetes	32	0.3%
Cancer history	52	0.4%
Hepatitis C antibody	101	0.9%
Cause of renal failure	0	0%
KRT start date	0	0%
Initial KRT type	0	0%
Place of dialysis	19	0.2%
Late referral	90	0.8%

Supplementary Table 2. Cross-tabulation of identification of Aboriginal and/or Torres Strait Islander people from ANZDATA and identification using ERA from nine linked NSW administrative health datasets

	**ERA**		
**ANZDATA**	Non-indigenous	Aboriginal and/or Torres Strait Islander people	**Total**
**Aboriginal and/or Torres Strait Islander people**	20	464	484
Row %	4.1%	95.9%	100%
**Non-indigenous**	10,995	229	11,224
Row %	98.0%	2.0%	100%
**Total**	11,015	693	11,708
Row %	94.1%	5.9%	100%

Supplementary Table 3. Accuracy of reporting of Aboriginal and/or Torres Strait Islander identification using ANZDATA augmented with ERA from the Admitted Patient Data Collection, compared to Enhanced Reporting of Aboriginality (ERA) from nine linked NSW administrative health datasets as the reference standard

	ANZDATA & APDC ERA	ERA (MHLL)	Sensitivity (95% CI)	Specificity (95% CI)	Positive predictive value (95% CI)	Negative predictive value (95% CI)
Overall	677	693	94.8 (92.9 – 96.3)	99.8 (99.7 – 99.9)	97.0 (95.5 – 98.2)	99.7 (99.5 – 99.8)
Sex						
Females	306	315	94.9 (91.9 – 97.1)	99.8 (99.6 – 99.9)	97.7 (95.3 – 99.1)	99.6 (99.3 – 99.8)
Males	371	378	94.7 (91.9 – 96.7)	99.8 (99.7 – 99.9)	96.5 (94.1 – 98.1)	99.7 (99.6 – 99.8)
Age (years)						
Under 18	20	20	100.0 (83.2 – 100.0)	100.0 (97.6 – 100.0)	100.0 (83.2 – 100.0)	100.0 (97.6 – 100.0)
18-44	129	129	96.9 (92.3 – 99.1)	99.7 (99.3 – 99.9)	96.9 (92.3 – 99.1)	99.7 (99.3 – 99.9)
45-64	369	382	93.7 (90.8 – 95.9)	99.7 (99.5 – 99.9)	97.0 (94.7 – 98.5)	99.4 (99.1 – 99.6)
≥ 65	159	162	95.1 (90.5 – 97.8)	99.9 (99.8 – 100.0)	96.9 (92.8 – 99.0)	99.9 (99.7 – 99.9)
Socio-economic status[1]						
Most disadvantaged (Q1&2)	462	473	95.6 (93.3 – 97.2)	99.8 (99.6 – 99.9)	97.8 (96.1 – 99.0)	99.6 (99.4 – 99.7)
Average (Q3)	105	110	93.6 (87.3 – 97.4)	99.9 (99.6 – 100.0)	98.1 (93.3 – 99.8)	99.6 (99.2 – 99.8)
Least disadvantaged (Q4&5)	106	106	92.5 (85.7 – 96.7)	99.8 (99.6 – 99.9)	92.5 (85.7 – 96.7)	99.8 (99.6 – 99.9)
Residence						
Major city	292	303	91.1 (87.3 – 94.0)	99.8 (99.7 – 99.9)	94.5 (91.3 – 96.8)	99.7 (99.5 – 99.8)
Outside major city	383	388	97.7 (95.6 – 98.9)	99.8 (99.5 – 100.0)	99.0 (97.3 – 99.7)	99.6 (99.2 – 99.8)
Mental illness[2]						
Severe and persistent	54	59	89.8 (79.2 – 96.2)	99.8 (98.7 – 100.0)	98.1 (90.1 – 100.0)	98.6 (96.9 – 99.5)
Moderate	24	24	100.0 (85.8 – 100.0)	100.0 (99.4 – 100.0)	100.0 (85.8 – 100.0)	100.0 (99.4 – 100.0)
None	599	610	95.1 (93.1 – 96.7)	99.8 (99.7 – 99.9)	96.8 (95.1 – 98.1)	99.7 (99.6 – 99.8)
Mental healthcare accessed						
Any inpatient	84	87	96.6 (90.3 – 99.3)	100.0 (99.3 – 100.0)	100.0 (95.7 – 100.0)	99.5 (98.5 – 99.9)
Community only	108	115	91.3 (84.6 – 95.8)	99.8 (99.4 – 100.0)	97.2 (92.1 – 99.4)	99.3 (98.7 – 99.7)
Mental illness duration (years)						
0–2 years	122	126	95.2 (89.9 – 98.2)	99.9 (99.5 – 100.0)	98.4 (94.2 – 99.8)	99.6 (99.1 – 99.8)
Over 2 years	70	76	90.8 (81.9 – 96.2)	99.8 (99.0 – 100.0)	98.6 (92.3 – 100.0)	98.7 (97.4 – 99.5)
Physical co-morbidity[2]						
Diabetes	422	434	94.2 (91.6 – 96.2)	99.8 (99.6 – 99.9)	96.9 (94.8 – 98.3)	99.5 (99.3 – 99.7)
Cardiovascular disease[3]	409	419	94.7 (92.2 – 96.7)	99.8 (99.6 – 99.9)	97.1 (94.9 – 98.5)	99.6 (99.4 – 99.8)
Other	259	265	94.7 (91.3 – 97.1)	99.8 (99.5 – 99.9)	96.9 (94.0 – 98.7)	99.6 (99.3 – 99.8)
Co-morbidity count[2]						
None	85	88	94.3 (87.2 – 98.1)	99.9 (99.7 – 100.0)	97.6 (91.8 – 99.7)	99.8 (99.5 – 99.9)
1–2	372	377	95.2 (92.6 – 97.1)	99.8 (99.7 – 99.9)	96.5 (94.1 – 98.1)	99.7 (99.6 – 99.8)
≥ 3	220	228	94.3 (90.4 – 96.9)	99.7 (99.4 – 99.9)	97.7 (94.8 – 99.3)	99.3 (98.8 – 99.6)

[1] SES quintiles one and two were grouped together, and quintiles four and five were grouped together

[2] Pre-existing at start of KRT

[3] Cardiovascular and cerebrovascular diseases = coronary artery disease, cerebrovascular disease, peripheral vascular disease

Supplementary Table 4. Accuracy of reporting of Aboriginality in ANZDATA compared to the Enhanced Reporting of Aboriginality (ERA) algorithm applied to the Admitted Patient Data Collection alone as the reference standard

	ANZDATA	ERA (APDC)	Sensitivity (95% CI)	Specificity (95% CI)	Positive predictive value (95% CI)	Negative predictive value (95% CI)
Overall	484	654	70.5 (66.8 – 74.0)	99.8 (99.7 – 99.9)	95.2 (93.0 – 97.0)	98.3 (98.0 – 98.5)
Sex						
Females	230	298	74.5 (69.2 – 79.3)	99.8 (99.6 – 99.9)	96.5 (93.3 – 98.5)	98.1 (97.6 – 98.5)
Males	254	356	67.1 (62.0 – 72.0)	99.8 (99.7 – 99.9)	94.1 (90.4 – 96.7)	98.4 (98.1 – 98.7)
Age (years)						
Under 18	9	20	45.0 (23.1 – 68.5)	100.0 (97.6 – 100.0)	100.0 (66.4 – 100.0)	93.3 (88.2 – 96.6)
18-44	89	125	68.0 (59.1 – 76.1)	99.7 (99.3 – 99.9)	95.5 (88.9 – 98.8)	97.2 (96.1 – 98.0)
45-64	281	357	75.4 (70.5 – 79.7)	99.7 (99.5 – 99.8)	95.7 (92.7 – 97.8)	97.8 (97.3 – 98.2)
≥ 65	105	152	64.5 (56.3 – 72.1)	99.9 (99.7 – 99.9)	93.3 (86.7 – 97.3)	99.0 (98.8 – 99.3)
Socio-economic status[1]						
Most disadvantaged (Q1&2)	335	451	71.8 (67.4 – 75.9)	99.8 (99.6 – 99.9)	96.7 (94.2 – 98.3)	97.6 (97.1 – 98.0)
Average (Q3)	79	102	74.5 (64.9 – 82.6)	99.8 (99.5 – 100.0)	96.2 (89.3 – 99.2)	98.6 (97.9 – 99.1)
Least disadvantaged (Q4&5)	68	97	60.8 (50.4 – 70.6)	99.8 (99.6 – 99.9)	86.8 (76.4 – 93.8)	99.1 (98.7 – 99.3)
Residence						
Major city	179	274	58.8 (52.7 – 64.6)	99.8 (99.7 – 99.9)	89.9 (84.6 – 93.9)	98.7 (98.5 – 98.9)
Outside major city	304	378	79.1 (74.6 – 83.1)	99.8 (99.5 – 99.9)	98.4 (96.2 – 99.5)	96.6 (95.8 – 97.3)
Mental illness[2]						
Severe and persistent	38	52	69.2 (54.9 – 81.3)	99.5 (98.3 – 99.9)	94.7 (82.3 – 99.4)	96.3 (94.1 – 97.9)
Moderate	20	24	83.3 (62.6 – 95.3)	100.0 (99.4 – 100.0)	100.0 (83.2 – 100.0)	99.3 (98.3 – 99.8)
None	426	578	70.1 (66.2 – 73.8)	99.8 (99.7 – 99.9)	95.1 (92.6 – 96.9)	98.3 (98.0 – 98.5)
Mental healthcare accessed						
Any inpatient	66	82	78.0 (67.5 – 86.4)	99.6 (98.7 – 100.0)	97.0 (89.5 – 99.6)	96.9 (95.2 – 98.2)
Community only	73	104	66.3 (56.4 – 75.3)	99.7 (99.3 – 99.9)	94.5 (86.6 – 98.5)	97.6 (96.7 – 98.3)
Mental illness duration (years)						
0–2 years	88	118	71.2 (62.1 – 79.2)	99.7 (99.3 – 99.9)	95.5 (88.8 – 98.7)	97.7 (96.8 – 98.4)
Over 2 years	51	68	72.1 (59.9 – 82.3)	99.6 (98.7 – 100.0)	96.1 (86.5 – 99.5)	96.7 (94.9 – 98.0)
Physical co-morbidity[2]						
Diabetes	342	406	80.3 (76.1 – 84.1)	99.7 (99.5 – 99.8)	95.3 (92.5 – 97.3)	98.5 (98.1 – 98.8)
Cardiovascular and cerebrovascular disease[3]	318	394	76.9 (72.4 – 81.0)	99.7 (99.6 – 99.8)	95.3 (92.3 – 97.3)	98.4 (98.0 – 98.7)
Other	202	250	77.2 (71.5 – 82.3)	99.7 (99.5 – 99.9)	95.5 (91.7 – 97.9)	98.4 (97.9 – 98.8)
Co-morbidity count[2]						
None	44	83	50.6 (39.4 – 61.8)	99.9 (99.7 – 100.0)	95.5 (84.5 – 99.4)	98.1 (97.5 – 98.7)
1-2	256	359	67.7 (62.6 – 72.5)	99.8 (99.7 – 99.9)	94.9 (91.5 – 97.3)	98.4 (98.1 – 98.7)
≥ 3	184	212	83.0 (77.3 – 87.8)	99.6 (99.1 – 99.8)	95.7 (91.6 – 98.1)	98.1 (97.3 – 98.6)

[1] SES quintiles one and two were grouped together, and quintiles four and five were grouped together

[2] Pre-existing at start of KRT

[3] Cardiovascular and cerebrovascular diseases = coronary artery disease, cerebrovascular disease, peripheral vascular disease

Supplementary Table 5. Accuracy of reporting of Aboriginal and Torres Strait Islander identification in the ANZDATA compared to identification based on the most recent record in the Admitted Patient Data Collection as the reference standard

	ANZDATA	APDC last record	Sensitivity (95% CI)	Specificity (95% CI)	Positive predictive value (95% CI)	Negative predictive value (95% CI)
Overall	484	609	75.0 (71.4 – 78.4)	99.8 (99.6 – 99.8)	94.4 (92.0 – 96.3)	98.6 (98.4 – 98.9)
Sex						
Females	230	281	78.3 (73.0 – 83.0)	99.7 (99.5 – 99.9)	95.7 (92.1 – 97.9)	98.5 (98.1 – 98.8)
Males	254	328	72.3 (67.1 – 77.0)	99.8 (99.6 – 99.9)	93.3 (89.5 – 96.1)	98.7 (98.4 – 99.0)
Age (years)						
Under 18	9	16	56.3 (29.9 – 80.2)	100.0 (97.7 – 100.0)	100.0 (66.4 – 100.0)	95.7 (91.4 – 98.3)
18-44	89	119	70.6 (61.5 – 78.6)	99.6 (99.2 – 99.9)	94.4 (87.4 – 98.2)	97.5 (96.6 – 98.3)
45-64	281	337	79.8 (75.1 – 84.0)	99.7 (99.5 – 99.8)	95.7 (92.7 – 97.8)	98.3 (97.8 – 98.7)
≥ 65	105	137	69.3 (60.9 – 76.9)	99.8 (99.7 – 99.9)	90.5 (83.2 – 95.3)	99.3 (99.0 – 99.5)
Socio-economic status[1]						
Most disadvantaged (Q1&2)	335	423	76.1 (71.8 – 80.1)	99.7 (99.6 – 99.9)	96.1 (93.5 – 97.9)	98.1 (97.7 – 98.4)
Average (Q3)	79	97	77.3 (67.7 – 85.2)	99.8 (99.4 – 99.9)	94.9 (87.5 – 98.6)	98.8 (98.2 – 99.2)
Least disadvantaged (Q4&5)	68	85	68.2 (57.2 – 77.9)	99.8 (99.6 – 99.9)	85.3 (74.6 – 92.7)	99.3 (99.0 – 99.6)
Residence						
Major city	179	249	63.9 (57.6 – 69.8)	99.8 (99.6 – 99.9)	88.8 (83.3 – 93.0)	99.0 (98.7 – 99.2)
Outside major city	304	358	83.0 (78.7 – 86.7)	99.7 (99.4 – 99.9)	97.7 (95.3 – 99.1)	97.4 (96.7 – 98.0)
Mental illness[2]						
Severe and persistent	38	49	73.5 (58.9 – 85.1)	99.5 (98.3 – 99.9)	94.7 (82.3 – 99.4)	97.0 (94.9 – 98.4)
Moderate	20	23	82.6 (61.2 – 95.0)	99.8 (99.0 – 100.0)	95.0 (75.1 – 99.9)	99.3 (98.3 – 99.8)
None	426	537	74.9 (71.0 – 78.5)	99.8 (99.6 – 99.8)	94.4 (91.7 – 96.4)	98.7 (98.4 – 98.9)
Mental healthcare accessed						
Any inpatient	66	75	84.0 (73.7 – 91.4)	99.5 (98.5 – 99.9)	95.5 (87.3 – 99.1)	97.9 (96.4 – 98.9)
Community only	73	100	69.0 (59.0 – 77.9)	99.7 (99.3 – 99.9)	94.5 (86.6 – 98.5)	97.9 (97.0 – 98.6)
Mental illness duration (years)						
0–2 years	88	111	74.8 (65.6 – 82.5)	99.7 (99.2 – 99.9)	94.3 (87.2 – 98.1)	98.1 (97.3 – 98.7)
Over 2 years	51	64	76.6 (64.3 – 86.2)	99.6 (98.7 – 100.0)	96.1 (86.5 – 99.5)	97.4 (95.7 – 98.5)
Physical co-morbidity[2]						
Diabetes	342	388	83.8 (79.7 – 87.3)	99.7 (99.5 – 99.8)	95.0 (92.2 – 97.1)	98.8 (98.5 – 99.1)
Cardiovascular and cerebrovascular disease[3]	318	370	81.4 (77.0 – 85.2)	99.7 (99.5 – 99.8)	94.7 (91.6 – 96.9)	98.8 (98.5 – 99.1)
Other	202	236	81.4 (75.8 – 86.1)	99.7 (99.5 – 99.9)	95.0 (91.1 – 97.6)	98.7 (98.3 – 99.1)
Co-morbidity count[2]						
None	44	73	56.2 (44.1 – 67.8)	99.9 (99.6 – 100.0)	93.2 (81.3 – 98.6)	98.6 (98.0 – 99.0)
1-2	256	332	72.3 (67.1 – 77.0)	99.8 (99.6 – 99.9)	93.8 (90.0 – 96.4)	98.7 (98.4 – 99.0)
≥ 3	184	204	86.3 (80.8 – 90.7)	99.6 (99.1 – 99.8)	95.7 (91.6 – 98.1)	98.5 (97.8 – 99.0)

[1] SES quintiles one and two were grouped together, and quintiles four and five were grouped together

[2] Pre-existing at start of KRT

[3] Cardiovascular and cerebrovascular diseases = coronary artery disease, cerebrovascular disease, peripheral vascular disease
